# Reproductive Toxicity: New Take on Perchlorate Effects

**DOI:** 10.1289/ehp.114-a637a

**Published:** 2006-11

**Authors:** Rebecca Renner

Perchlorate, an ingredient in solid rocket fuel, is known to affect thyroid function by blocking iodine uptake, disrupting physical growth and neurological development. A new fish study in the August 2006 issue of *Environmental Toxicology and Chemistry* indicates that perchlorate may also disrupt sexual development by overmasculinizing both males and females.

In the multiyear project, Richard Bernhardt, a graduate student at the University of Alaska Anchorage, and two colleagues caught wild three-spine sticklebacks, a tiny fish species often used in toxicology. For three weeks they kept the fish in water treated with 30, 60, or 100 ppm perchlorate. After the adults spawned, the scientists raised the offspring to maturity in similarly treated water.

At least half of the offspring in all the treatment groups and more than 70% of those in the highest treatment group died. Many of the surviving male offspring in all treatment groups failed to develop “nuptial coloration” (bright blue and red colors that signal they are ready for spawning), ignored females, and displayed no courtship behaviors such as nest building or attentiveness to prospective mates. Three fish looked and behaved like males but became ripe with eggs; these fish turned out to be genetically female. Cellular analysis of the genetic females’ gonads revealed that the organs developed as a mixture of egg-producing and sperm-producing tissues (“ovotestes”). Genetically male fish also developed abnormally large testes. All of the treated fish grew more slowly than untreated controls.

“We saw reproductive effects because we treated the fish during critical developmental windows after conception and analyzed the fish for reproductive effects after they reached sexual maturity,” says Bernhardt. The mechanisms that produced these effects remain unclear.

Biologist Helen Jordinson (née Crane) of the Environment Agency in England and Wales reported in the April 2005 issue of *EHP* that perchlorate exposure delayed growth in fathead minnows exposed for 28 days from embryos. “The [Bernhardt et al.] paper is very interesting,” she says, though she urges caution in interpreting the findings. “Significant toxicity was also reported at the same perchlorate concentrations as the behavioral and pathological effects,” she explains, which could indicate that effects resulted from the overall stress caused by such high perchlorate concentrations and not from a specific mode of action unique to perchlorate.

Jordinson also notes that thyroid hormones in fish have been shown to vary depending on the stage of maturation of the gonads and whether spawning is occurring, indicating links between the thyroid gland and the reproductive system. Thyroid receptors have also been found on the gonads of some species. “It is therefore possible that the reproductive effects [of perchlorate] could be due to disruption of the thyroid system in the sticklebacks,” she says.

The lowest perchlorate dose in the experiments was more than 1,000 times higher than the EPA’s suggested limit of 24.5 ppb for perchlorate in drinking water. Bernhardt says that the concentrations are lower than groundwater concentrations at several contaminated sites in the United States. Perchlorate has been found at ppb levels in drinking water, whereas concentrations reported in food vary from low ppb concentrations to highs of several ppm.

Next Bernhardt plans to study sticklebacks’ dose response to perchlorate with the help of a glycoprotein glue called spiggin. During the breeding season, males produce spiggin for use in building their nests. Just as researchers use the protein vitellogenin as a biomarker for feminizing effects, Bernhardt and colleagues plan to use spiggin as a marker for masculinization.

## Figures and Tables

**Figure:**
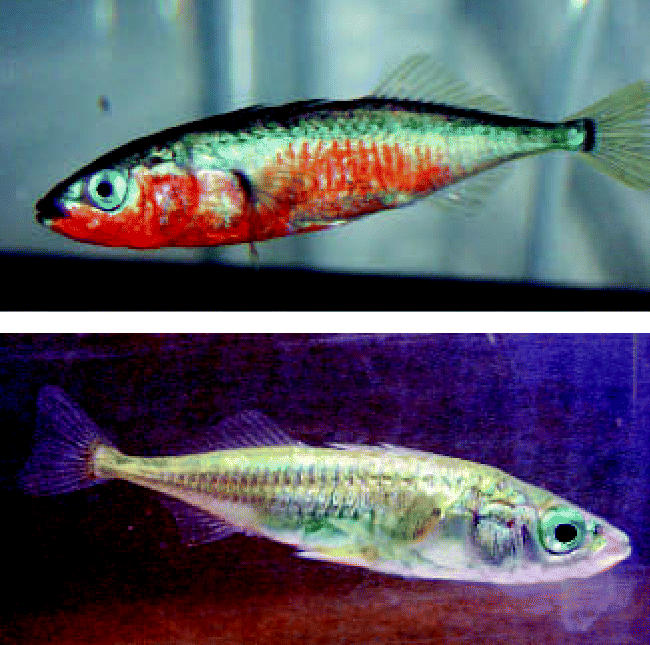
Failure to shine Male threespine sticklebacks normally undergo a dramatic color change (top) to signal readiness to spawn. Perchlorate-treated male fish (above) failed to “color up,” ignored females, and displayed no courtship behaviors.

